# Magnetically Induced Catalysis: Definition, Advances, and Potential

**DOI:** 10.1002/anie.202424151

**Published:** 2025-04-18

**Authors:** A. Bordet, W. Leitner, B. Chaudret

**Affiliations:** ^1^ Max Planck Institute for Chemical Energy Conversion Stiftstraße 34–36 45470 Mülheim an der Ruhr Germany; ^2^ Université de Toulouse, INSA, LPCNO Laboratoire de Physique et Chimie des Nano‐Objets, CNRS, UMR 5215 135 Avenue de Rangueil Toulouse 31077 France; ^3^ Institute for Technical and Macromolecular Chemistry RWTH Aachen University 52074 Aachen Germany

**Keywords:** Adaptivity, Localized heating, *Magnetically induced catalysis*, Magnetocatalysis, Rapid heating

## Abstract

The rapidly growing importance of electrification in the chemical industry opens room for disruptive innovations regarding energy input into catalytic processes. Energy efficiency and dynamics of renewable energy supplies represent important challenges, but the design of catalytic systems to cope with such new frameworks may also stimulate the discovery of new catalyst materials and reaction pathways. In this context, many opportunities arise when catalysts are activated in a rapid, localized, and energy‐efficient manner. Among the various concepts to achieve adaptivity in catalysis, magnetic induction heating applied directly at the catalyst or in vicinity of the active site has gained increasing attention recently. In this Scientific Perspective, we provide a coherent framework to the emerging field of catalysis using magnetic fields—and in particular alternating current magnetic fields—to activate catalytic materials and define it as ***magnetically induced catalysis***. Promising approaches and selected examples are described to illustrate the scientific concept and to highlight its broad potential for innovation in catalysis from laboratory to industrial scale.

## Graphical abstract

In this scientific perspective, we intend to provide a coherent framework for the field of ***magnetocatalysis*** by highlighting opportunities associated with the use of magnetic fields to activate catalysts. A particular focus will be placed on ***magnetically induced catalysis***, providing an overview on current advances and identifying scientific and technical challenges on the path toward a widespread use of this technology in research and industry.

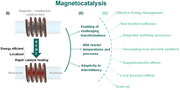

## 1 Introduction

The dynamic deployment of cost‐effective technologies generating electricity from renewable sources is driving the increasing *decarbonization* of the electricity sector providing major opportunities for the electrification of the chemical industry toward the *defossilization* of the chemical value chain.^[^ ^1^, ^2^, ^3^ ^]^ Although the operation of catalytic processes still relies mainly on fossil‐based process heat,^[^ ^4^, ^5^, ^6^ ^]^ its electrification is highly desirable to limit greenhouse gases emissions and meet the growing environmental and societal expectations for products with low‐carbon footprints. As a result, extensive efforts in science and technology are currently dedicated to the development of novel approaches for energy input into catalytic processes, including direct electrical heating, green hydrogen heating, electrocatalysis, plasma activation, mechanocatalysis, etc.^[^ ^7^, ^8^, ^9^, ^10^, ^11^, ^12^, ^13^ ^]^ A major challenge is associated with the intermittency of renewable electricity, which can also be turned into an opportunity if the processes allow for rapid reaction to availability and price in the grid system.^[^ ^14^, ^15^, ^16^, ^17^ ^]^

**Figure 1:**
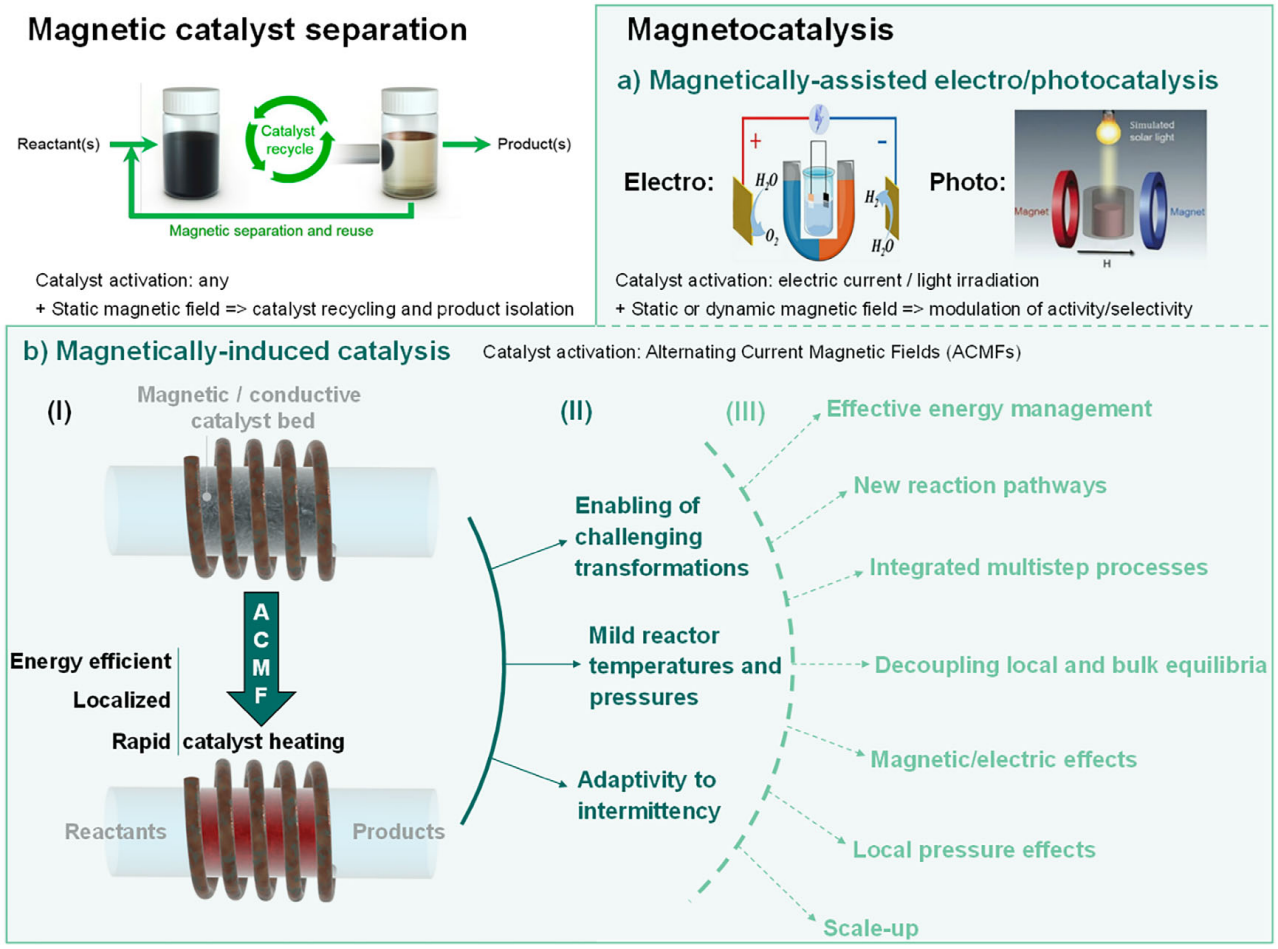
Main applications of magnetic fields in catalysis, including catalyst separation (illustration adapted from Ref. [36]) and reactivity induction and modulation in magnetocatalysis. a) Reactivity modulation in magnetic field‐assisted electro/photocatalysis (illustrations adapted from Refs. [33] and [35]), and b) reactivity induction in magnetically induced catalysis, illustrated with its principles (Part I), state of the art with demonstrated benefits (Part II), and potential opportunities (Part III), as discussed step‐by‐step in the following sections of this article.

In this context, magnetic induction heating offers an attractive potential, as proven in a range of robust technologies. Magnetic induction is being used for decades in industry (e.g., metallurgy) and private appliances (e.g., domestic cooktops) owing to its proven superior energy efficiency as compared to other heating methods.^[^ ^18^, ^19^, ^20^ ^]^ Paradoxically, magnetic induction heating has so far only been scarcely studied in catalysis.^[^ ^21^, ^22^, ^23^ ^]^ Historically, magnetic materials received attention in the field of catalysis for their ability to be easily separated from reaction mixtures through the application of a static external magnetic field, i.e., of a permanent magnet. The use of magnetic nanoparticles or magnetic support materials enabled the easy recovery and reuse of catalysts for a wide variety of transformations and facilitated the isolation of target products (Figure 1).^[^ ^24^, ^25^, ^26^, ^27^ ^]^ A large number of examples have been reported in the literature from this field of research that is still dynamic nowadays^[^ ^28^, ^29^ ^]^ and has been reviewed recently.^[^ ^30^, ^31^ ^]^ In contrast, ***magnetocatalysis*** can be defined as a general term for the ***application of magnetic fields to induce or modulate catalytic activity and selectivity***. For example, modulating the reactivity of catalysts activated by electric current or light irradiation through the application of magnetic fields is an intensively investigated approach (cf. magnetically assisted electro/photocatalysis, Figure 1a).^[^ ^32^, ^33^, ^34^, ^35^ ^]^ Within the broad definition of magnetocatalysis, this perspective will focus on the emerging field *of* ***magnetically induced catalysis*** *, which involves the* ***application of alternating current magnetic fields (ACMFs) to activate and control catalytic materials***. Although the activation by ACMFs is related to localized and specific heating in most cases, we do not restrict the induction to only thermal effects at this stage.

In this respect, methods using magnetic induction for heating of reactors^[^ ^37^, ^38^, ^39^, ^40^ ^]^ will not be discussed in the present perspective. In these technologies, the heating is provided to bulk metallic parts such as the reactor walls and not selectively induced to catalysts and catalytically active materials. Indeed, the magnetic induction activation of catalysts directly offers attractive key features beyond energy efficiency. The contactless and selective heating provided by magnetic induction is of potential interest to directly heat catalysts in a localized manner, thereby precluding the need for heating whole reactor systems, including reactor walls, solvents, etc. Thus, the local temperature at the catalyst material and the active site can exceed the bulk temperature of the reactor system largely, often by several tens of degrees and more. The magnetic induction‐based thermal energy transfer is also extremely rapid,^[^ ^18^ ^]^ potentially enabling reversible switching of catalytic states in terms of activity and selectivity as required to unlock adaptivity to intermittent electricity supply.^[^ ^17^ ^]^

However, despite great promises, the true potential and practicability of magnetic induction heating and activation in catalytic processes is still elusive, and many fundamental and practical questions remain. Its potential for the opening of new reaction pathways, for the conversion of complex raw materials, for the integration of reaction and separation steps, as well as for the combination of steps through new tandem approaches is still to be revealed.

In this scientific perspective, we intend to provide a coherent framework for the field of ***magnetically induced catalysis*** (Figure 1b) by highlighting opportunities associated with the use of ACMFs to activate catalysts. We will provide an overview on current advances and identify scientific and technical challenges on the path toward a widespread use of this technology in research and industry.

## 2 Part (I): Principles of Magnetically Induced Catalysis

### 2.1 Definition

In the past decade, several research groups have been exploring the potential of magnetic induction activation in *catalysis*, and each group essentially adopted its own terminology to refer to this technology and to describe ACMF parameters. For example, the following descriptions have been used through the years to describe the use of ACMFs to enable catalytic reactions:

1. “*Inductive Heating in Synthesis*,” with ACMFs described by a *frequency* in *kHz* and a field *amplitude* in *parts* per *thousand (ppt)*.^[^ ^41^ ^]^
2. “*Radio Frequency Heating in Catalysis*,” with ACMFs described by a *frequency* in *kHz* and a *heat generation rate* in *kW* *m* ^−3^.^[^ ^42^ ^]^
3. “***Magnetically Induced Catalysis***,” with ACMFs described by a *frequency* in *kHz* and a field *amplitude* in *millitesla (mT)*.^[^ ^43^, ^44^ ^]^
4. “*Direct Hysteresis Heating in Catalysis*,” with ACMFs described by a *frequency* in *kHz* and a field *amplitude* in *millitesla (mT)*.^[^ ^45^ ^]^
5. “*Magnetic Induction Heating in Catalysis*,” with ACMFs described by a field *amplitude* in *millitesla (mT)*.^[^ ^46^ ^]^
6. “*Radio Frequency Induction Heating in Catalysis*,” with ACMFs described by a *frequency* in *kHz* and a *maximum voltage* in *kV*.^[^ ^47^ ^]^

Here, we intend to introduce a common general terminology for catalytic transformations enabled by materials exposed to magnetic fields and, in particular, to ACMFs: ***magnetically induced catalysis***, with the aim to establish a coherent framework alongside fields such as electrocatalysis, photocatalysis, microwave catalysis, plasma catalysis, or mechanocatalysis. *In this context, we propose to standardize the parameters and units used to describe ACMFs by systematically providing the field frequency and amplitude in SI units (kHz and mT, respectively)*. All examples discussed in this review have been normalized accordingly where possible.

### 2.2 Mechanisms and Efficiency of Magnetic Induction Heating

**Figure 2:**
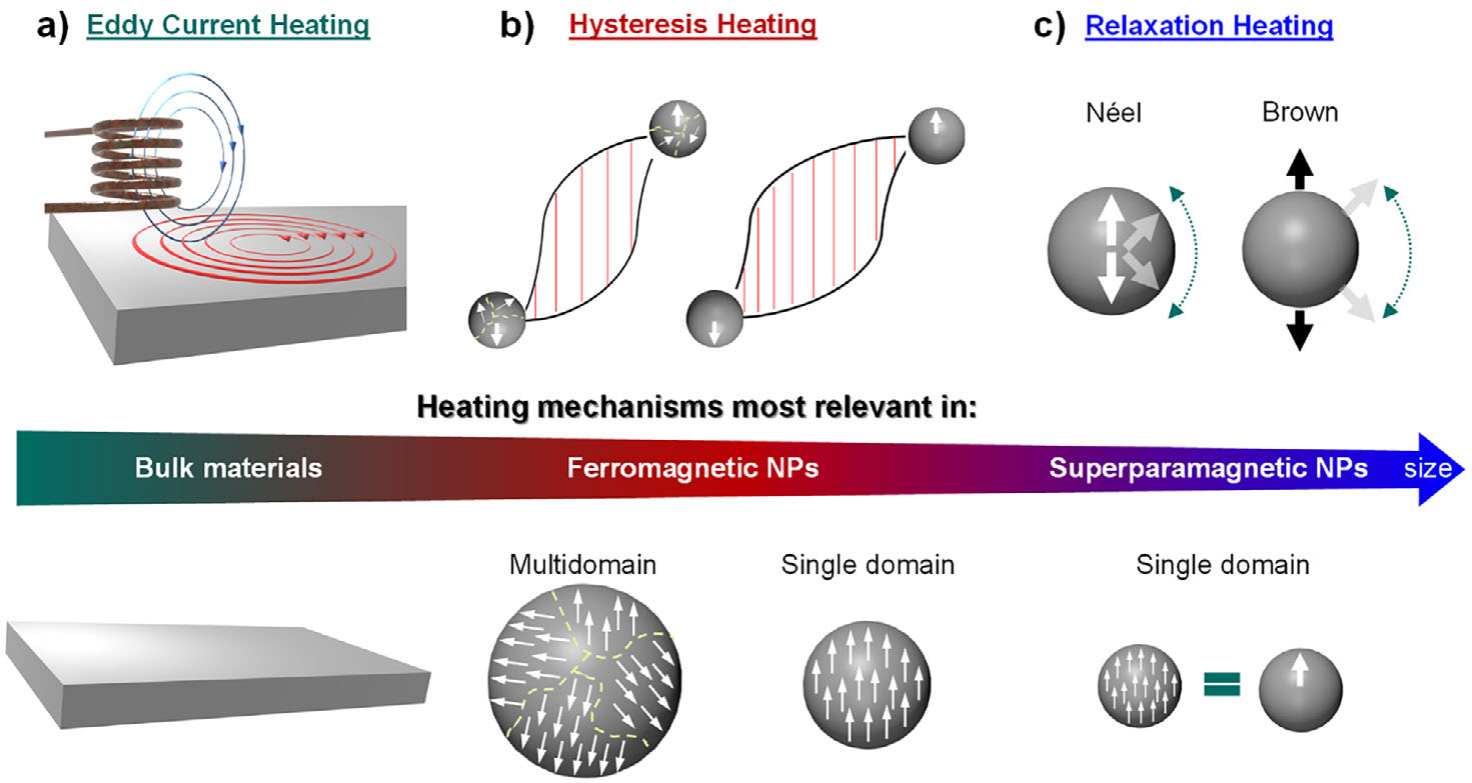
Illustration of the different heating mechanisms occurring in materials exposed to ACMFs. a) Eddy current heating, b) hysteresis heating, and c) relaxation heating. N.B. While the dominance of a heating mechanism depends on the material's size, several heating mechanisms can coexist. The color gradient of the arrow illustrates which mechanisms are most relevant in which material type while reflecting the possibility of coexisting heating mechanisms.

The conversion of magnetic energy into thermal energy by a magnetic material exposed to an ACMF can be described according to the following main mechanisms: 1) Eddy current heating, 2) hysteresis losses, and 3) relaxation/susceptibility losses (Figure 2).^[^ ^48^, ^49^, ^50^ ^]^

***Eddy currents*** are induced in electrically conductive materials exposed to ACMFs, resulting in the Joule heating of the materials (Figure 2a). The efficiency of heating by induced eddy current depends on the electrical resistivity and geometry of the material, as well as on the ACMF frequency. Eddy current heating is particularly used for the heating of ***bulk conductive materials*** (e.g., in metallurgy and in domestic kitchen induction hobs) and can also be relevant for susceptors in the millimeter and micrometer scale. It becomes negligible for materials at the nanoscale, owing to the section dependency of eddy current loops (a minimum target material thickness of three skin depths is typically indicated for viable eddy current heating^[^ ^51^ ^]^) as well as to the rather low conductivity of most magnetic nanoparticles.

***Hysteresis heating*** is typical and dominant for ***ferromagnetic and ferrimagnetic particles*** placed in ACMFs and is due to an irreversible magnetization process (Figure 2b). Energy losses (i.e., heat production) can originate from two main mechanisms dependent on the particle size: i) Particles that are larger than the critical diameter (material dependent) possess multiple magnetic domains, and hysteresis heating proceeds through displacement of the magnetic domain walls; ii) below the critical diameter, single‐domain magnetic particles have no domain walls and hysteresis heating originates from the rotation of the particles’ magnetic moments. ***Single‐domain ferromagnetic nanoparticles*** were shown to maximize the hysteresis loop area and thus hysteresis heating efficiency. NPs in the size range between the single domain and the multidomain states are called pseudo‐single domain NPs and can combine heating mechanisms.^[^ ^52^ ^]^ This configuration was found beneficial for the hysteresis heating of magnetite NPs.^[^ ^53^ ^]^

Below a certain size, single‐domain ferromagnetic nanoparticles adopt a superparamagnetic behavior, meaning that magnetism is not observed in the absence of an external magnetic field. For ***superparamagnetic NPs***, heating under ACMFs occurs mainly through energy losses from the ***relaxation*** of the magnetization upon removal of the external magnetic field (Figure 2c). Néel relaxation corresponds to the rotation of the atomic magnetic moments within immobile NPs, while Brown relaxation describes the physical rotation of the NPs. While both relaxation mechanisms can proceed simultaneously, Brown relaxation can only occur for NPs in a liquid environment.

Magnetic induction heating is a contactless method that transfers energy directly and selectively to a target material with minimal wasted heat/energy. The energy efficiency of magnetic induction heating has been extensively studied and compared to that of other methods for technical heating applications such as metallurgy, Czochralski crystal growth, zone refining in semiconductor manufacturing, or domestic cooktops.^[^ ^18^, ^19^, ^20^ ^]^ Magnetic induction heating was found to be systematically and very significantly more energy efficient than conventional electric (resistive) and gas‐fired heating, with power savings at constant delivered power ranging from 15 to 45%.^[^ ^18^, ^19^, ^20^, ^54^ ^]^

For such applications, the parts to be heated are bulk materials, and the heating is provided by induced eddy currents. While direct efficiency measurements and comparisons between heating mechanisms across scales can be complex, the specific absorption rate (SAR) of magnetic particles can be determined to describe materials’ heating power (in W g^−1^). The SAR corresponds to the power that 1 g of magnetic particles can adsorb and release as heat. Table 1 provides examples of the SAR of selected materials that can be heated by eddy currents, hysteresis, and relaxation.

**Table 1:** Selected examples of materials used in magnetic induction heating, including particle size, main heating mechanism, and SAR.

Material	Particle size (nm)	Magnetic domain	Dominant heating mechanism	*μ*_0_H (mT)	SAR at 100 kHz (W g^−1^)	Reference
Fe wool	Bulk	Multi	Eddy current	47	69	[55]
Fe_3_O_4_	130	Multi	Hysteresis	60	82	[56]
Fe_3_O_4_	97	Single	Hysteresis	23	6.6	[53]
Fe_3_O_4_	125	Pseudo‐single	Hysteresis	23	8.1	[53]
Fe_3_O_4_@Fe_3_C	70	Multi	Hysteresis	33	150	[57]
Zn_0.4_Fe_2.6_O_4_@CoFe_2_O_4_	120	Multi	Hysteresis	47	600	[58]
Zn_0.4_Fe_2.6_O_4_@CoFe_2_O_4_	60	Multi	Hysteresis	47	841	[58]
Co_0.7_Fe_2.3_O_4_	23	Multi	Hysteresis	40	343	[59]
CoNi	30	Multi	Hysteresis	55	150	[60]
Fe(0)	14	Single	Hysteresis	47	1095	[43]
Fe(0)@Fe_5_C_2_	13.5	Single	Hysteresis	47	648	[61]
ICNPs	15	Single	Hysteresis	47	3220	[43]
Co_0.7_Fe_2.3_O_4_	16	Single	Hysteresis	40	857	[59]
γ Fe_2_O_3_	7.5	Single	Relaxation	31	29	[62]
γ Fe_2_O_3_	16.5	Single	Relaxation	31	236	[62]
Fe_3_O_4_	11	Single	Relaxation	31	112	[63]
Fe_30_Ni_70_	17	Single	Relaxation	47	645	[64]

Interestingly, SAR values are much higher for nanoparticles heated by hysteresis or relaxation mechanisms than for bulk materials heated by eddy currents. Thus, while superior heating efficiency through induced eddy currents has been demonstrated over conventional methods, the magnetic heating of nanoparticles has the potential of being even more efficient. Overall, highest heating power is typically observed for single domain ferromagnetic nanoparticles, as exemplified by iron carbide nanoparticles (ICNPs) reaching 3 kW g^−1^ (Entry 11).

### 2.3 Practical Aspects

ACMF generators are commercially available at a variety of suppliers. Of particular importance when selecting an ACMF generator are the frequency (*f*) and amplitude (*μ*_0_H) ranges accessible and the corresponding power (*P*). For lab‐scale experiments using magnetic nanoparticles as heating agents, selecting these parameters in the ranges [*f* = 50–400 kHz], [*μ*_0_H = 10–100 mT], and [*P* = 1–10 kW] is suitable. Typically, *f* is fixed while *μ*_0_H can be easily modulated. Commercial equipment generally comes with standard water‐cooled copper coils of various potential geometries. Air‐cooled systems with copper or Litz‐wire coils are also becoming increasingly available and were shown to be more energy efficient. Coil geometry, field parameters, and generator power need to be sized to the magnetic material considered, intended transformation, and reaction scale.

The use of magnetic or electrically conductive materials should be avoided in the design of reactors for magnetically induced catalysis to prevent the generation of eddy currents that can lead to bulk reactor heating, energy losses, thermal stress, reduced mechanical strength, and component failure. Insulating thermally and chemically stable materials should be used instead, such as borosilicate glass, quartz, ceramics, and some polymers (e.g., PTFE, PEEK). For liquid‐phase batch reactions, mixing can be ensured by convection, overhead mechanical stirring, or solvent circulation. Fixed‐bed plug‐flow reactors are suitable for the exploration of magnetically induced catalysis under continuous flow conditions.

For reactions in the liquid phase, the choice of solvent was shown to be particularly important. Depending on the targeted reaction, the boiling point of the solvent can be selected near the surface temperature of the catalyst to prevent hampering transport of reactants to the active sites through the formation of a gas layer (Leidenfrost effect),^[^ ^65^ ^]^ or much higher to carry out transformations involving sensitive materials.

## 3 Part (II): State of the Art

### 3.1 Localized Heating

**Figure 3:**
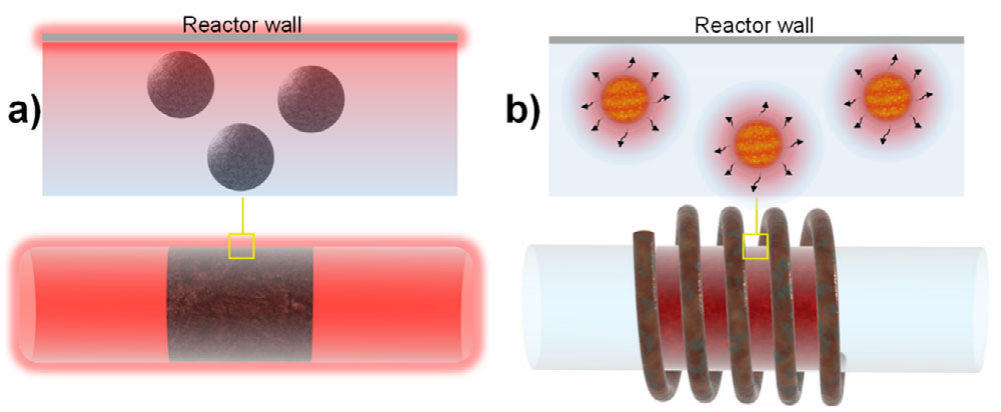
Illustration of the heat distribution in reactors where catalysts are heated a) conventionally and b) by magnetic induction.

ACMFs induce thermal energy in magnetic materials in a contactless and selective manner. When considering a reaction catalyzed by a magnetic material, this means that the heat required to unlock activity is generated directly at the site(s) where the elementary processes of the catalytic cycle occur (Figure 3).

This extremely localized thermal energy generation obviates the heating of reactor parts and reaction medium, thereby offering promising perspectives for energy savings beside the intrinsic high energy efficiency of magnetic induction heating discussed earlier. In addition, localized catalyst heating can potentially lead to temperature gradients in reactors and thus enable high‐temperature transformations under mild conditions observable in the bulk phase. The potential of localized heating by magnetic induction has been explored in catalysis by several groups in the past decade.^[^ ^41^, ^42^, ^43^, ^44^, ^45^, ^46^, ^47^, ^55^, ^60^, ^61^, ^64^, ^65^, ^66^, ^67^, ^68^, ^69^, ^70^, ^71^, ^72^, ^73^, ^74^, ^75^, ^76^, ^77^ ^]^

For reactions in the ***gas phase***, magnetically induced catalysis was pioneered by Meffre et al., who studied Fischer–Tropsch syntheses using iron‐based NPs heated by magnetic induction.^[^ ^66^ ^]^ The authors showed that 11–13 nm Fe, Fe@FeCo, and Fe@Ru NPs could be selectively heated using ACMFs generated by an air‐cooled Litz wire coil to catalyze the hydrogenation of CO to a mixture of alkanes (methane, ethane, propane, and butane).

**Figure 4:**
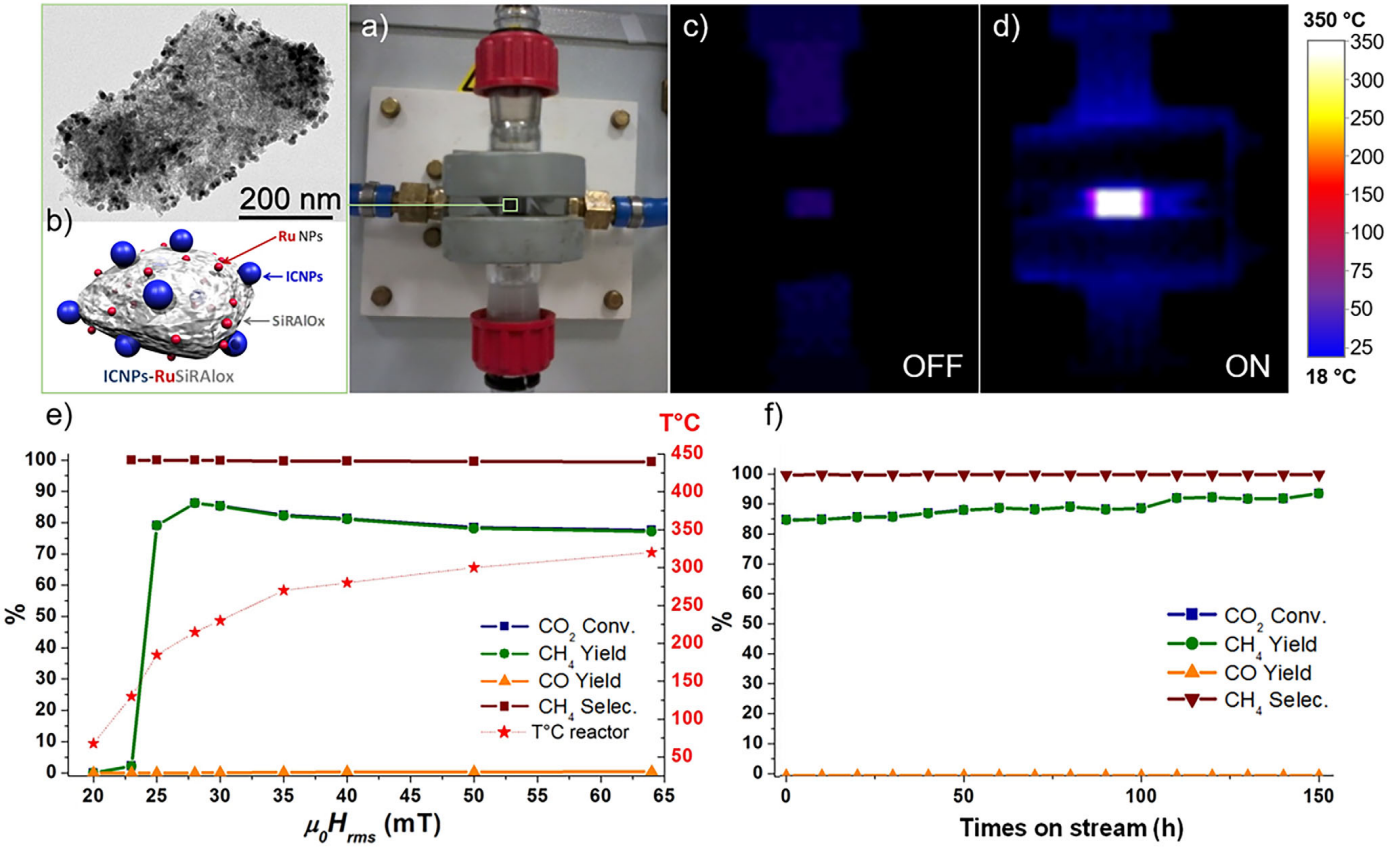
Magnetically induced CO_2_ methanation using ICNPs‐RuSiRAlOx as catalysts. a)–d) Continuous flow reactor containing ICNPs‐RuSiRAlOx demonstrating localized heating through IR camera imaging, e) catalytic performance as a function of the field amplitude, and f) catalytic performance over time at 28 mT and 300 kHz. Adapted from Ref. [43].

Later, Bordet et al. from the same group reported the preparation of 15 nm ICNPs possessing record heating power (SAR values up to 3000 W g^−1^ at 100 kHz, 47 mT).^[^ ^43^ ^]^ ICNPs were applied to the continuous flow hydrogenation of CO_2_ at atmospheric pressure, giving CO and methane as main products (Figure 4). Immobilizing ICNPs on SiRAlOx and using them as heating agents for small Ru NPs resulted in a spectacular activity and selectivity enhancements, with methane yields as high as 93% with >99% selectivity (28 mT, 300 kHz, 1071 L h^−1^ g Ru^−1^, Figure 3e). Such performance could be preserved for 150 h on stream without any decrease in heating power or catalytic performance (Figure 4f). This study demonstrated the possibility to perform magnetically induced catalysis in the gas phase under continuous flow conditions.^[^ ^43^ ^]^

In this case, the localized catalyst heating by magnetic induction was monitored using an infrared camera, evidencing temperatures up to 350 °C at the external surface of the glass reactor in direct contact with the catalyst bed, while other neighboring reactor parts were close to room temperature (Figure 4d). Although this provides a clear and valuable macroscopic visualization of the selective catalyst heating, such measurements do not provide information on the working temperature at the surface of the catalyst itself. In a follow‐up study, the authors incorporated a thermocouple inside their reactor to determine more accurately the temperature in the catalyst bed.^[^ ^68^ ^]^

**Figure 5:**
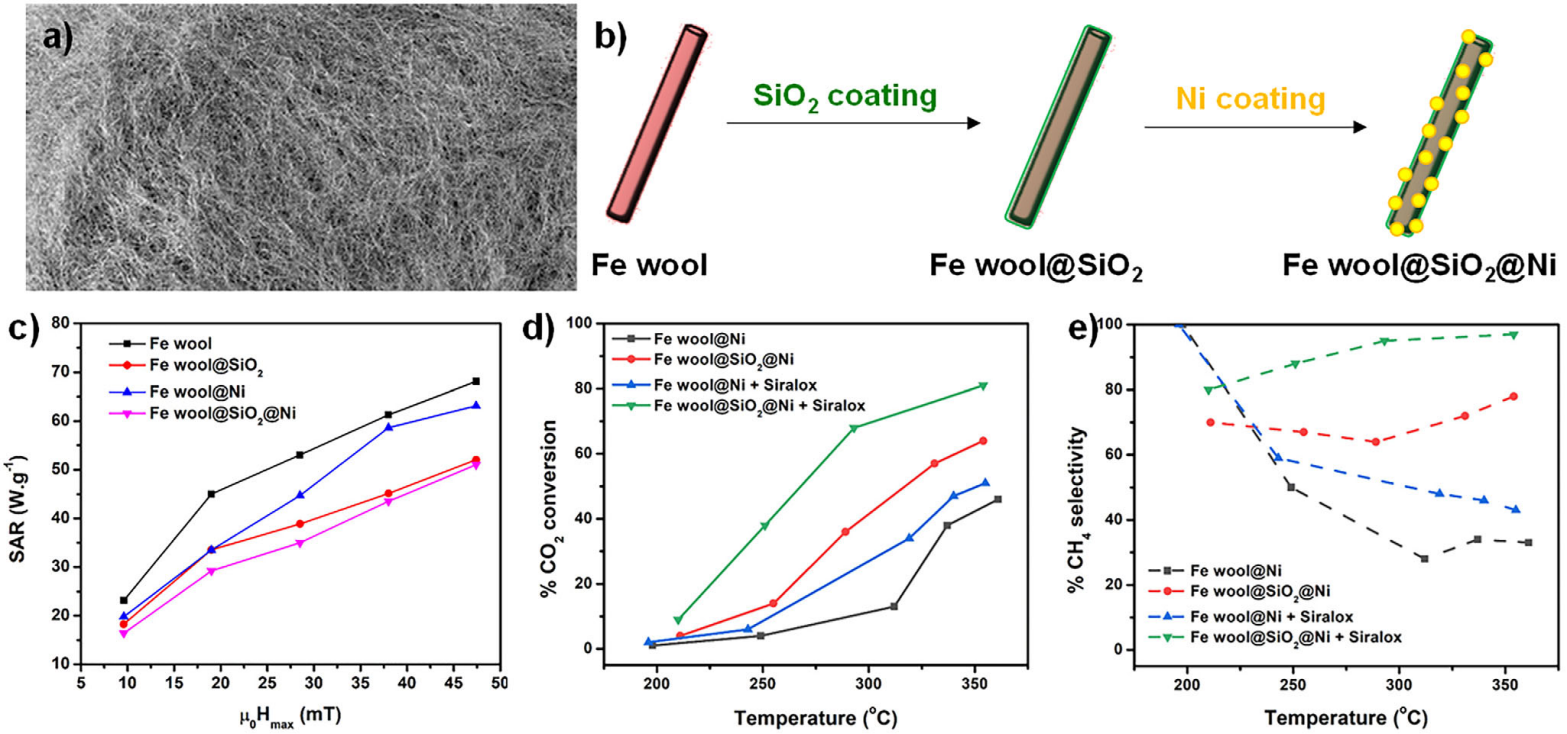
Magnetically induced methanation of CO_2_ using iron wool coated with SiO_2_ and Ni. a) Picture of the iron wool. b) Scheme of the coating process. c) SAR as a function of the ACMF amplitude at 100 kHz. d) CO_2_ conversion as a function of temperature. e) CH_4_ selectivity as a function of temperature. Adapted from Ref. [55].

Recently, the possibility to use commercial and inexpensive iron wool as magnetically responsive support material for magnetically induced catalysis was explored by Ghosh et al. (Figure 5).^[^ ^55^ ^]^ In particular, Fe wool was coated with a SiO_2_ layer (40–60 nm thickness) and used for the deposition of a Ni film (40–60 nm thickness). Exposure of the resulting Fe_wool_@SiO_2_@Ni to ACMFs (100 kHz, 47 mT) resulted in the heating of the material by eddy currents and unlocked activity for the hydrogenation of CO_2_ to methane (64% conversion, 78% selectivity to methane at ca. 355 °C). The presence of SiRAlOx as a filler in the catalyst bed further stabilized the Fe_wool_@SiO_2_@Ni material, enhancing its performance (81% conversion, 90% selectivity, 0.42 mol_CH4_ g_Ni_^−1^ h^−1^).

The physical mixing of a classical catalyst and a cheap heating element can also offer a possibility to scale up reactions more easily.^[^ ^71^, ^78^, ^79^, ^80^ ^]^ In this respect, using pure iron wool associated with a classical methanation catalyst (Ni/SirAlox or Ni/Al_2_O_3_) allowed to reach a good conversion (85%–95%) to a total selectivity and a high energy efficiency for the hydrogenation of CO_2_ into methane.^[^ ^72^ ^]^

Vinum and coworkers applied cobalt‐nickel NPs (ca. 35 nm) heated by magnetic induction to steam methane reforming (32 mT, 69 kHz).^[^ ^60^ ^]^ CoNi NPs were heated up to ca. 800 °C and were found to be active for the production of hydrogen. Interestingly, for this endothermic transformation, the rate of induction‐heated catalysts was limited by chemical reactivity, while conventional heating catalysts were limited by heat transfer. In this case, the temperature was measured at the reactor outlet using a Type‐K thermocouple.

**Figure 6:**
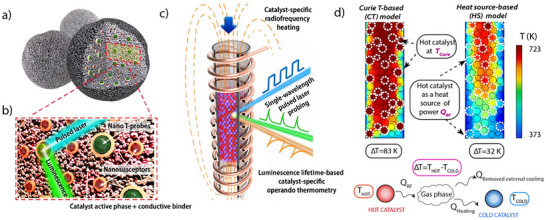
a) Schematic representation of the multifunctional design of consolidated catalyst bodies incorporating additional auxiliary magnetic susception and thermophosphor sensing functionalities. b) Blown‐up view of the cross‐sectional area marked with the red frame in panel a). Ferromagnetic nanocrystals (brown polyhedra) and lanthanide‐based thermophosphor nanoparticles (green, multigrain spheres) appear coated by a continuous SiO_2_ overlay. c) Schematic depiction of a packed‐bed reactor inserted in a RF coil and filled with a blend of consolidated bodies of two catalyst materials between which a steady temperature gradient is established by catalyst‐specific heating. The first catalyst material features ferromagnetic susceptors and thus self‐heats via induction mechanisms (red particles). The second “cold” catalyst material lacks ferromagnetic susception and it is mainly heated via convective mechanisms (purple particles). Temperature is monitored via operando luminescence thermometry. d) Computer fluid dynamics (CFD)‐derived temperature profiles obtained for a model reactor assuming two scenarios for the heating of the hot catalyst. Reproduced from Ref. [70] with permission from the Royal Society of Chemistry.

Interestingly, Farpón et al. reported the preparation of a multicomponent catalytic system assembled from magnetic NPs (25 nm CoFe_2_O_4_ NPs coated with SiO_2_, heating agent), thermophosphor functionalities (Tb‐ and Eu‐doped Y_2_O_3_ NPs (ca. 300 nm) embedded in SiO_2_), an ethene dimerization catalyst (Ni NPs on aluminosilicate), and an olefin metathesis catalyst (Re NPs on Y‐zeolite) (Figure 6).^[^ ^70^ ^]^ The temperature gradient generated by the locally heated magnetic NPs was used to activate neighboring Ni and Re catalysts at different temperatures, thus allowing the tandem dimerization (Ni catalyst at 137 °C) and metathesis (Re catalyst at 87 °C) of ethene to propene, two reactions steps that are typically carried out separately due to their temperature incompatibility. Interestingly, the incorporated thermophosphore functionalities enabled contactless luminescence thermometry that provided information on temperature levels in relatively close vicinity to the catalytically active sites. Computational fluid dynamics (CFD) heat transport simulations supported the possibility to establish temperature differences between two solid catalysts activated by ACMFs and blended in a gas–solid reactor. As shown in Figure 6d, simulations based on Curie temperature‐based and heat source‐based models predict a permanent temperature difference between the “hot” (magnetically activated) and “cold” catalysts, while the magnitude of the temperature gradient depends on various operational settings (e.g., ACMF parameters, reactor cooling, catalyst volume ratio, etc.).

Selected examples of magnetically induced catalysis in the gas phase are provided in Table 2.

**Table 2:** Examples of magnetically induced catalysis in the gas phase.

Reaction	Catalyst	*f* (kHz)	*μ*_0_H (mT)	Reactor type	*T*_Reactor_ (°C)	Reference
Fischer–Tropsch	Fe@Ru	54	50	Batch	n.d.	[66]
Fischer–Tropsch	Fe@FeCo	–	–	–	–	[66]
CO_2_ methanation	ICNPs@Ru‐SiRAlOx	300	28	Flow	350^a)^	[43]
CO_2_ hydrogenation	ICNPs	300	35	Batch	n.d.	[67]
CO_2_ methanation	Ferrites + Ni/SiRAlOx	300	53	Flow	450^b)^	[69]
CO_2_ methanation	Ni/OCF	n.d.	n.d.	Flow	320^b)^	[81]
CO_2_ methanation	Ni/metal oxides	100	12	Flow	220^b)^	[82]
CO_2_ methanation	Fe_wool_@SiO_2_@Ni	100	40	Flow	300–350^b)^	[55]
Steam methane reforming	NiCo	32	70	Flow	800^b)^	[60]
Steam methane reforming	NiCo	189	40	Flow	800^b)^	[83]
Propane dehydrogenation	FeCo@C (+Ni or Pt‐Sn)	300	40–60	Flow	400–600^b)^	[84]
CO oxidation	Pt@Fe_3_O_4_	205	27	Flow	220^c)^	[46]

^a)^ Determined by IR camera/pyrometer.

^b)^ Determined by a thermocouple.

^c)^ Determined by an optic fiber probe.

Application of magnetically induced catalysis to reactions in the ***liquid phase*** was first described by Ceylan and coworkers, who used silica‐coated superparamagnetic iron oxide nanoparticles heated by magnetic induction through relaxation mechanisms to catalyze various organic transformations (e.g., transesterification, condensation, Claisen rearrangement, etc.) under continuous flow conditions.^[^ ^41^ ^]^ Reaction temperatures (60–170 °C range) were determined by an infrared pyrometer reflecting the global surface temperature of the reactor.

**Figure 7:**
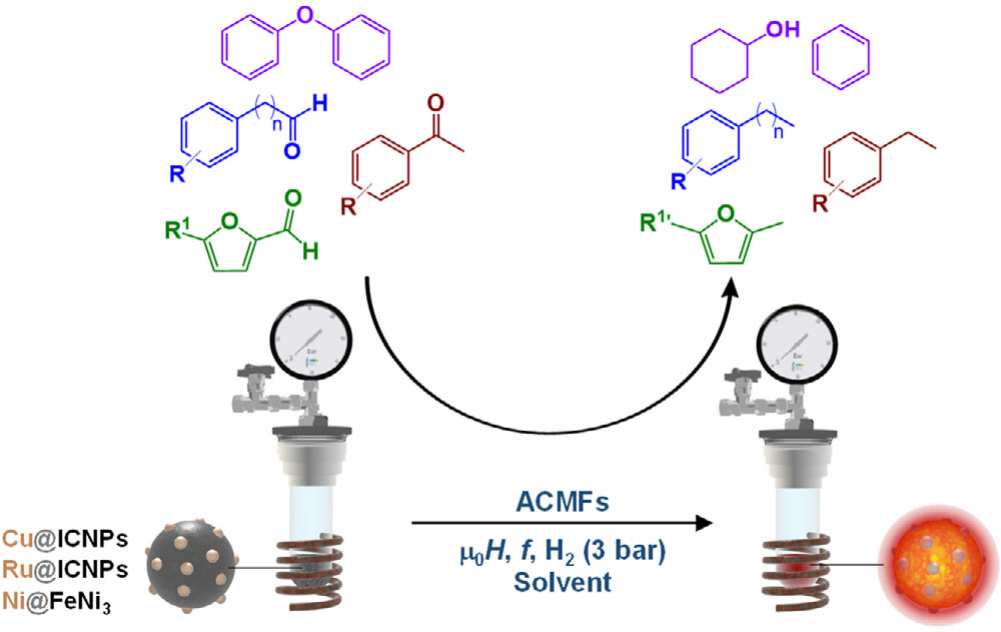
Magnetically induced hydrogenation, hydrodeoxygenation, and hydrogenolysis reactions using magnetic nanoparticle catalysts.

More recently, a few groups reported the use of tailor‐made ferromagnetic (e.g., Ru@ICNPs, Cu@ICNPs, etc.) or superparamagnetic (e.g., FeNi, etc.) nanoparticle catalysts to perform difficult reactions in solutions (Figure 7).^[^ ^72^, ^73^, ^74^, ^75^, ^76^, ^85^ ^]^ One well‐studied example is the hydrodeoxygenation (HDO) of ketones and aldehydes often containing aromatic moieties. These reactions usually performed at high temperature and pressure in heterogeneous catalysis (50 bar H_2_, *T *> 200 °C) readily occur under magnetic induction under apparent mild conditions (3 bar H_2_, solvent temperature 100–150 °C). This is likely due to a catalyst temperature well above the mean solvent temperature and, possibly to the build‐up of a pressure belt around the catalytic particle. This has been for example demonstrated when comparing the HDO of acetophenone in classical thermal catalysis or in magnetically induced catalysis using Ru@ICNPs. The conclusion was that the catalytic NPs could reach a temperature of 236 °C in toluene, the mean temperature of which remained close to 110 °C.^[^ ^72^ ^]^ Mustieles Marin and coworkers prepared bimetallic FeNi NPs (FeNi_3_ and FeNi_3_@Ni, ca. 16 nm) that they applied to the magnetically induced hydrodeoxygenation and hydrogenolysis of biomass‐model substrates such as acetophenone, furfural, and diphenylether (49 mT, 300 kHz, 3 bar H_2_).^[^ ^74^ ^]^ Under ACMF, IR camera measurements determined a temperature of 150–160 °C on the external wall of the Fisher–Porter bottle used as reactor. Under these conditions, furfural was fully converted, with 37% selectivity toward methylfuran. Interestingly, performing the same reaction under conventional heating at 170 °C led to lower conversion (60%) and selectivity (8%), suggesting that the local temperature at the surface of the magnetically heated NPs is much higher than that of their global environment measured by IR camera. Along the same line, Lin et al. showed that copper‐decorated ICNPs (ICNPs@Cu) are excellent catalysts for the magnetically induced hydrodeoxygenation of aromatic aldehydes to aromatic alkanes under unusually mild conditions (3 bar H_2_, 100 °C global temperature).^[^ ^75^ ^]^ The authors demonstrated that ICNPs@Cu generate local hot spots allowing the reaction to proceed at very high temperature (250–280 °C) while the global observable conditions remained mild (100 °C). In this case, the surface temperature of the NP catalysts was estimated by monitoring the boiling of a series of solvents of known boiling points upon exposure of ICNPs@Cu to the ACMF. These selected examples evidence the possibility to carry out difficult reactions in solution using ***magnetically induced catalysis***.

Another interest of the “hot spots” is linked to reactions requiring a high energy of activation but involving thermally sensitive reagents or products. In this respect, it has been recently shown that alcohol amination could be achieved with a high conversion and selectivity using CoNi@Cu nanoparticles heated magnetically (300 kHz, 49 mT, 150 °C global temperature), whereas the same reaction carried out thermally (160 °C) only led to a poor conversion and a low selectivity.^[^ ^86^ ^]^

**Figure 8:**
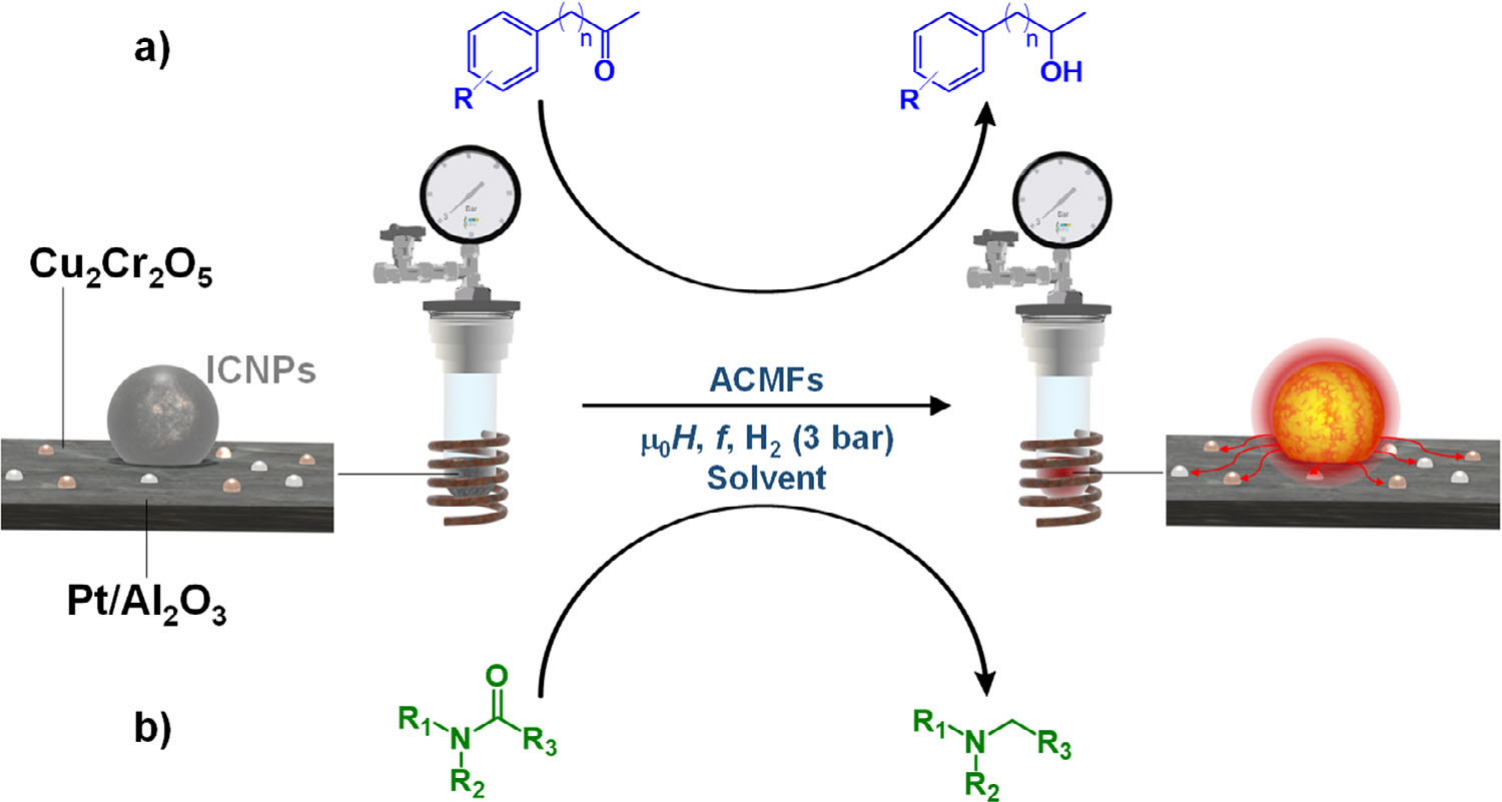
Magnetically induced hydrogenation of a) ketones and b) amides using standard commercial heterogeneous catalysts heated by ICNPs.

Kreissl et al. reported an alternative catalyst design, in which magnetic NPs are immobilized on standard commercial heterogeneous catalysts, thereby granting them with magnetic activation capabilities. This new approach to magnetically induced catalysis was demonstrated through the preparation of a multifunctional hydrogenation catalyst composed of commercial copper chromite Cu_2_Cr_2_O_5_ functionalized with ferromagnetic ICNPs (Figure 8).^[^ ^44^ ^]^ ICNPs were selectively heated by magnetic induction, transferring the thermal energy to the catalytic Cu_2_Cr_2_O_5_ material that also acted as support. The ICNPs@Cu_2_Cr_2_O_5_ catalyst was applied to the magnetically induced batch and flow hydrogenation of aromatic ketones, whereby ICNPs effectively activated the Cu_2_Cr_2_O_5_ surface without affecting its intrinsic selectivity. Aromatic ketones were effectively and selectively hydrogenated to aromatic alcohols under conditions (70 mT, 350 kHz, 3 bar H_2_, 80 °C global temperature as measured by IR camera) much milder than what is required for a Cu_2_Cr_2_O_5_ catalyst heated conventionally. Most recently, the same authors expanded this strategy and demonstrated the possibility to immobilize ICNPs on standard commercial Pt/Al_2_O_3_ catalysts (Figure 8).^[^ ^65^ ^]^ Under ACMF (70 mT, 350 kHz), ICNPs play the role of heating agent for small Pt NPs in their vicinity. This local and intense thermal energy transfer enabled efficient hydrogenation of amides to amines under unprecedented mild conditions (1–3 bar H_2_, 150 °C global temperature as measured by IR camera).^[^ ^65^ ^]^ Estimation of the catalyst temperature through the solvent boiling approach showed surface temperatures lying in the 290–330 °C range. Interestingly, comparable catalytic performances were out of reach through conventional heating at similar temperatures, even at higher H_2_ pressures up to 50 bar. Conductive support materials can also be heated by Eddy currents to heat immobilized NPs, as shown by Wang et al. for the methanation of CO_2_ using Ni NPs supported on magnetically heated carbon felt.^[^ ^81^ ^]^

The idea of a hot catalyst in a cold medium can also be used in electrochemistry where magnetically heating FeC@Ni nanoparticles was shown to decrease the overpotential of the oxygen evolution reaction by 200 mV.^[^ ^87^ ^]^ Selected examples of magnetically induced catalysis in the liquid phase are provided in Table 3.

**Table 3:** Examples of magnetically induced catalysis in the liquid phase.

Reaction	Catalyst	Solvent	*F* (kHz)	*μ*_0_H (mT)	Reactor type	*T*_Reactor_ ^a)^ (°C)	*T*_Catalyst_ (*T* °C)	Reference
Transesterification	SPION@SiO_2_	Toluene	25	235 ppt	Flow	60	n.d.	[41]
Heck coupling	Pd@SPION@SiO_2_	DMF/H_2_O	25	325 ppt	Flow	120	n.d.	[41]
Ketone hydrogenation	ICNPs@Cu_2_Cr_2_O_5_	Heptane	350	64	Batch and flow	80	160^b)^	[44]
Aldehyde HDO	Cu@ICNPs	Decalin	350	70	Batch	100	250–280^b)^	[75]
Aldehyde hydrogenation/HDO	FeCo@Ni@C	H_2_O	300	50–100	Batch	100	213–268^c)^	[76]
Ketone HDO	Ru@ICNPs	Mesitylene	300	58	Batch	150–160	n.d.	[72]
HDO/hydrogenolysis	FeNi	Mesitylene	300	49	Batch	150–160	n.d.	[74]
Lignin hydroprocessing	NiCo and Ru@NiCo	Decane	300	65	Batch	120–150	ca. 300^d)^	[85]
Alcohol amination	Co_4_Ni_6_@Cu	None	300	49	Batch	150	n.d.	[86]
Amide hydrogenation	ICNPs@Pt/Al_2_O_3_	Decalin	350	70	Batch	150	290–330^b)^	[65]

^a)^ Determined by IR camera/pyrometer.

^b)^ Estimated via the solvent boiling approach.

^c)^ Estimated via the activation energy approach.

^d)^ Speculated based on reaction mechanism.

### 3.2 Rapid Heating and Switchable Systems

**Figure 9:**
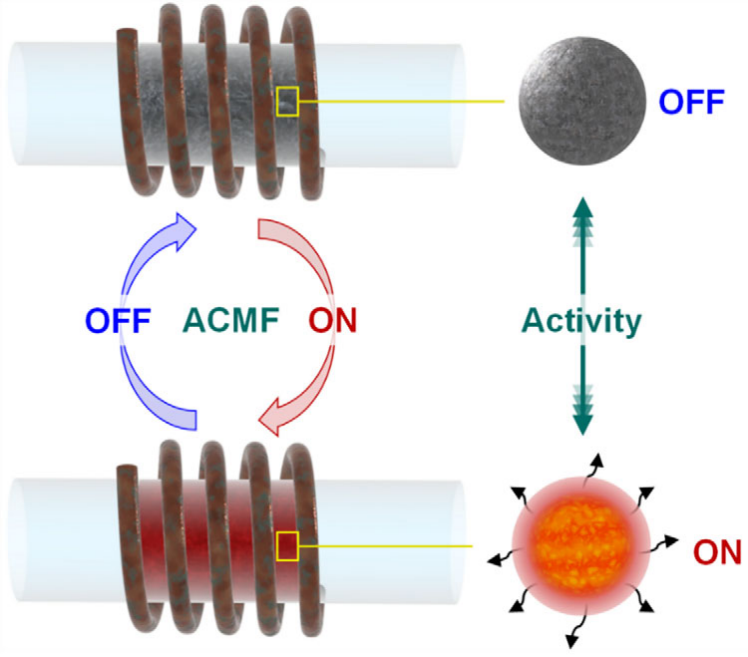
Illustration of the rapid catalyst heating and cooling provided by magnetic induction heating.

The thermal energy transfer to magnetic materials exposed to ACMFs is not only localized, it is also extremely rapid, especially at the nanoscale. Particles heated by magnetic induction were found to reach their working temperature in just a few milliseconds, which is considerably faster than with conventional heating methods.^[^ ^18^ ^]^ In catalysis, the rapid heating of catalysts can be of great interest to address the challenges associated with the use of renewable energy sources, as it has the potential to enable a very fast onset and stop of catalytic activity (Figure 9).^[^ ^17^ ^]^ Several groups have explored this potential and demonstrated the possibility to use ACMFs to switch catalytic activity ON or OFF in a very rapid and reversible manner to adapt to intermittent power supply.^[^ ^43^, ^44^, ^65^, ^75^, ^81^, ^84^, ^88^ ^]^

**Figure 10:**
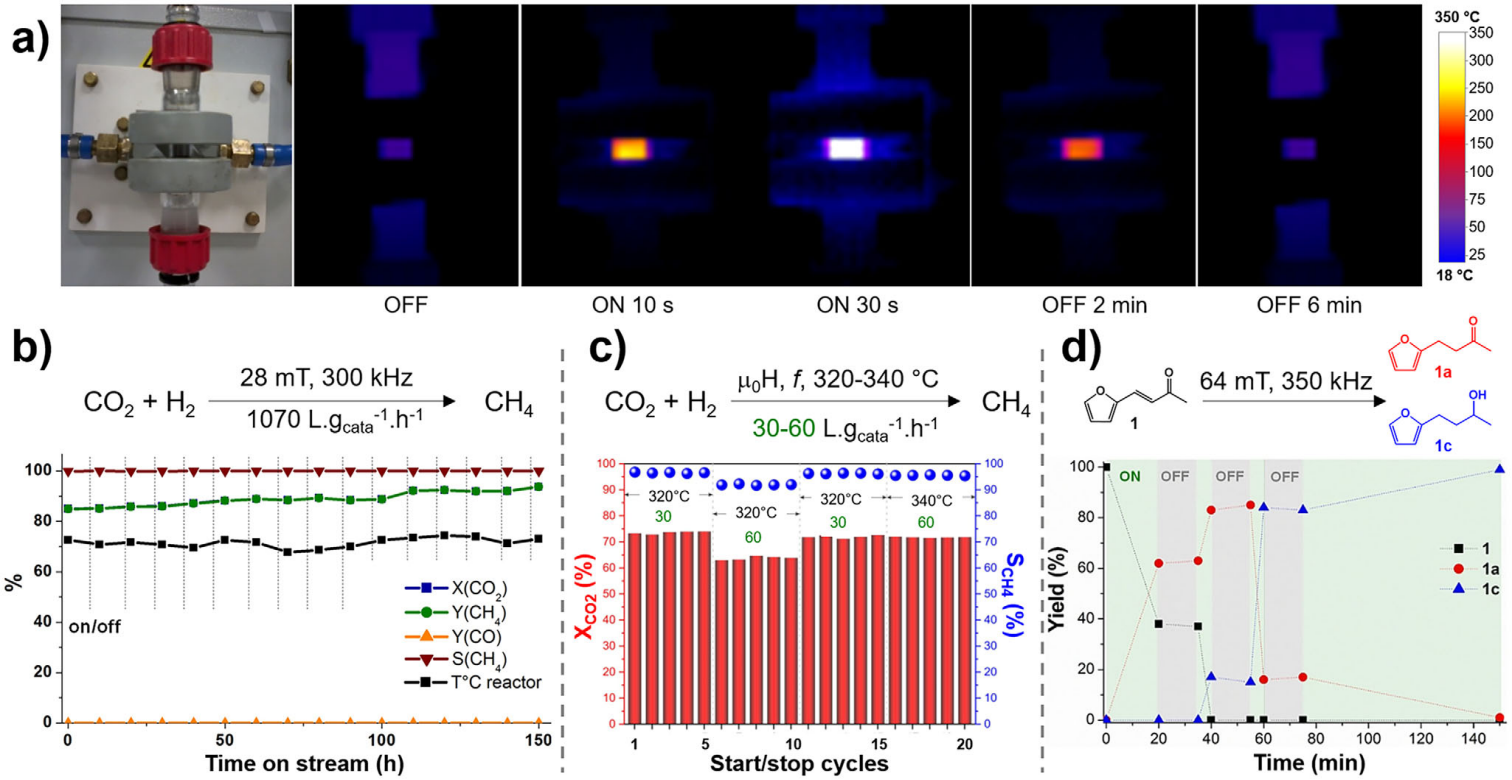
Examples of magnetically induced hydrogenation reactions demonstrating rapid, reversible, and robust ON‐OFF switching. a) Rapid heating and cooling (by simple heat dissipation) of ICNPs‐RuSiRAlOx catalysts by ON‐OFF switching of ACMFs, and b) associated catalytic performance in CO_2_ methanation. c) Magnetically induced CO_2_ methanation using nickel NPs–decorated carbon felt, and its response to ON‐OFF cycles and flow rates. d) Time profile of the magnetically induced hydrogenation of biomass‐derived furfuralacetone using an ICNPs@Cu_2_Cr_2_O_5_ catalyst, showing perfectly concomitant start and stop of the conversion upon switching the ACMF ON and OFF. Adapted from Refs. [43, 44, 81]

For example, Bordet et al. showed that turning ON the ACMF results in the very rapid heating of the ICNPs@Ru@SiRAlOx catalyst bed, which cools down quickly due to heat dissipation when the ACMF is turned OFF.^[^ ^43^ ^]^ Monitoring by IR camera revealed that the external surface of the glass reactor in contact with the catalyst bed reached more than 350 °C in 30 s upon turning the ACMF ON and cooled down to room temperature in about 6 min when the ACMF was turned OFF (Figure 10a). Start/stop cycles during CO_2_ methanation did not affect catalytic performance for over 150 h on stream, demonstrating the robustness of the ICNPs@Ru@SiRAlOx catalyst as well as its adaptivity to intermittent power supply (Figure 10b).

Wang and coworkers investigated the magnetically induced CO_2_ methanation under dynamic operation using Ni NPs immobilized on carbon felt.^[^ ^81^ ^]^ In this case, the electrically conductive carbon felt support is heated through eddy currents, and transfers the generated thermal energy to the catalytically active Ni NPs. The authors determined the global temperature of the reactor using a laser pyrometer, and observed best catalytic performance (ca. 75% methane yield at 30 L g_cat_^−1^ h^−1^) at 320 °C. Rapidly starting and stopping the ACMF while simultaneously replacing the H_2_/CO_2_ feed with helium resulted in rapid heating and cooling of the catalyst bed in the 320–260 °C range, and did not affect the catalyst performance even after several cycles (Figure 10c). Such start/stop cycles were not successful using conventional resistive heating due to longer heating and cooling times.

In the liquid phase, Kreissl et al. showed that using a ICNPs@Cu_2_Cr_2_O_5_ catalyst for the magnetically induced hydrogenation of aromatic ketones in batch and flow (70 mT, 350 kHz, 3 bar H_2_, 80 °C global temperature as measured by IR camera), turning ON or OFF the ACMF during the recording of a conversion time profile to mimic intermittency in electricity supply resulted in a perfectly concomitant switch of catalytic activity, demonstrating the catalyst's adaptivity (Figure 10d).^[^ ^75^ ^]^

While studies so far used the rapid heating provided by magnetic induction heating to access adaptivity to intermittent power supply, there may be also promising opportunities to apply it for rapid selectivity switches in reaction pathways involving various intermediates and products.

### 3.3 Temperature Determination

**Figure 11:**
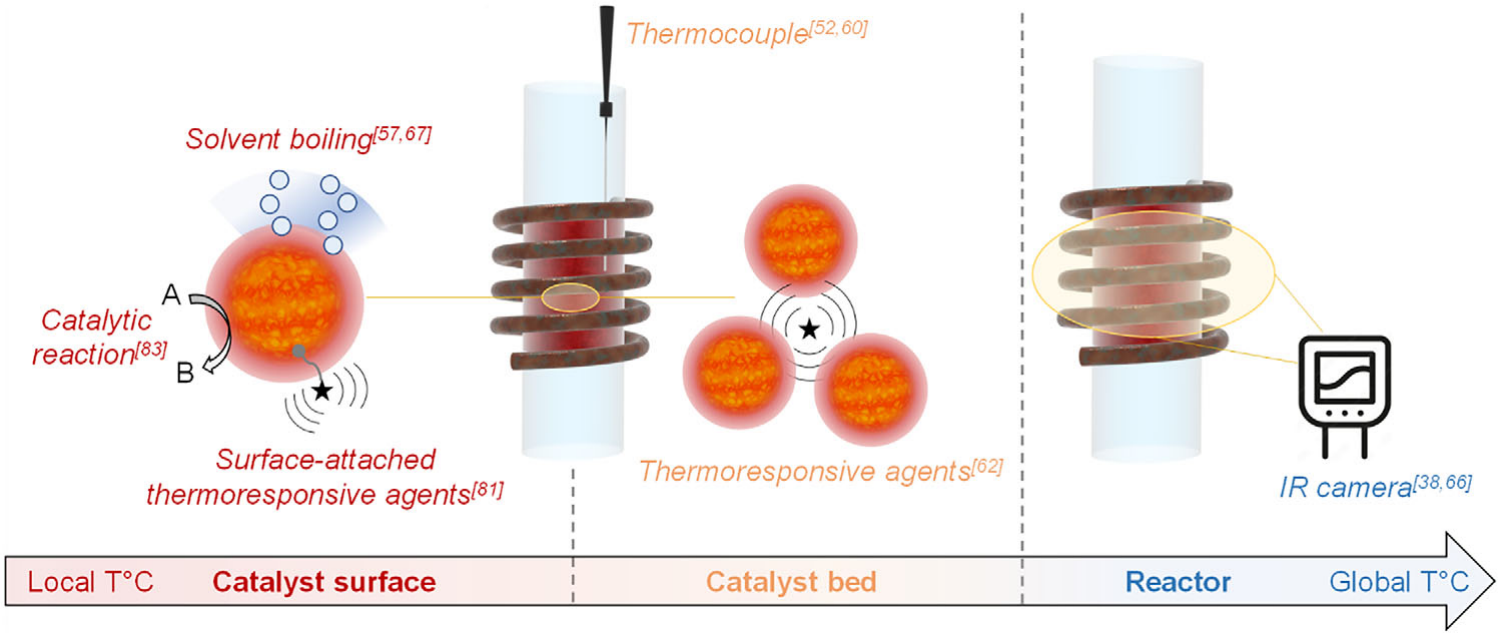
Illustration of the different approaches to temperature determination in magnetically induced catalysis.

The accurate determination of temperatures in magnetically induced catalysis is of crucial importance, and can be challenging due to technical restrictions associated with the use of ACMFs (e.g., no bulk conductive material should be inserted in catalytic reactors). As mentioned in above‐described examples, various strategies have been developed over the years in order to measure temperatures from the macroscale (i.e., reactor) to the mesoscale (i.e., close to catalyst surface) (Figure 11). In particular, IR sensors/cameras are useful to measure the global temperature distribution at the surface of reactors in a contactless manner.^[^ ^43^, ^74^ ^]^ Temperatures determined following this approach are systematically lower than the temperature within the catalyst bed due to radial energy losses. To determine temperatures within catalyst beds/reaction media, in‐bed thermocouples can be used.^[^ ^53^, ^60^, ^68^ ^]^ However, it is important to note that such in‐bed devices can be susceptible to ACMFs and heat by their own eddy current generation. Thus, proper selection or design of thermocouples is essential to limit this effect and prevent the recording of misleading results.^[^ ^53^ ^]^ Even upon application of appropriate thermocouples, one should be aware of the potential lag between the temperature measured by the thermocouple that is typically carried by the fluid temperature and the catalyst surface temperature.^[^ ^53^ ^]^

The incorporation of thermosensitive luminescent materials in catalyst beds or at the surface of magnetically heated NPs proved more accurate but requires a much more complex catalyst design/assembly.^[^ ^70^, ^89^ ^]^ Recently, Noble et al. reported an in‐situ magnetometry method to determine the temperature of magnetic particles heated by ACMFs.^[^ ^53^ ^]^ While elegant, this approach requires an accurate description of the materials’ physicochemical properties and does not take into consideration potential dynamic changes occurring during catalytic reactions. Interestingly, looking at catalytic performance through the determination of activation energies was proposed as a method to estimate the temperature of magnetically heated catalysts.^[^ ^90^ ^]^ Alternatively, monitoring of the boiling of a series of solvents of known boiling points was shown to be a very simple approach to estimate fairly accurately the surface temperature of magnetically heated material/catalyst.^[^ ^65^, ^75^ ^]^

### 3.4 Energy Efficiency

While the benefits associated with the localized and rapid catalyst heating by magnetic induction are being increasingly explored and convincingly described, the energy efficiency of catalytic reactors run by magnetic induction remains comparatively less studied.^[^ ^43^, ^47^, ^82^, ^83^, ^91^, ^92^, ^93^ ^]^ Energy efficiency is broadly defined as the *useful energy output* divided by the *total energy input* and was refined following two main approaches until now depending on the research question to be answered:

1. The ***heating energy efficiency*** is called here ***E*_H_**, which refers to the efficiency of the conversion of electrical energy into thermal energy through magnetic induction, as discussed in the introduction. It can be written as:
(1) $$E_{H}=\left(m_{\mathrm{Cat}}\times SAR_{\mathrm{Cat}}\right)/\left(P_{\text{ACMF generator}}+P_{\text{Cooling system}}\right)\times 100$$

2. The ***efficiency of the energy transfer*** called here ***E*_T_**, which refers to the transfer of electrical energy by magnetically induced processes into chemical energy stored in product molecules. It is particularly relevant when considering the production of energy carriers for chemical energy conversion.

Where *m*_Cat_ is the mass of catalyst (in g), SAR_Cat_ is the specific absorption rate of the catalyst (in W g^−1^), *P*_ACMF generator_ is the power consumed by the ACMF generator (in W), and *P*_Cooling system_ is the power consumed by the cooling system (in W).

It can be written as:

(2) $$E_{T}=\left(P_{\mathrm{Product}}\right)/\left(P_{\text{ACMF generator}}+P_{\text{Cooling}\;\text{system}}+P_{\mathrm{Reactants}}\right)\times 100$$

where *P*_Product_ is the power (in W) carried by the products generated in a magnetically induced reaction, *P*_ACMF generator_ is the power consumed by the ACMF generator (in W), *P*_Cooling system_ is the power consumed by the cooling system (in W), and *P*_Reactants_ is the power carried by the reactants (in W). *P*_Product_ and *P*_Reactants_ were so far derived from the gross calorific value (in J g^−1^) of the molecules considered (gases), which refers to the amount of energy generated through the combustion of 1 g of gas.

A possible reason for the rare determination of energy efficiencies may be that lab‐scale magnetic induction heating devices are designed to be flexible to offer the wide range of magnetic field amplitudes and frequencies required for testing and optimization and thus are not optimized to maximize energy efficiency.

For example, a pioneer study on CO_2_ methanation for which the quantity of catalyst, reactant flow, and coil geometry were not optimized reported an *E*_H_ of ca. 20% and an *E*_T_ (representing the energy carried by the output flow of methane as compared to the electricity and H_2_ flow input) of about 1%.^[^ ^43^ ^]^ However, the authors indicated that *E*_H_ and *E*_T_ up to 80% and 33%, respectively, could be potentially reached by maximizing the amount of catalyst and the reactant flow rate within the limits of the experimental set‐up.

In this context, Faure et al. investigated the energy efficiency of CO_2_ methanation performed by magnetic induction activation of physical mixtures of iron microparticles and Ni@CeO_2_ (10 wt% Ni loading).^[^ ^91^ ^]^ The authors showed that for low flow rates (<100 L h^−1^), the *E*_T_ is severely impacted by the coil's power consumption (ACMF generation and cooling). Changing from a conventional water‐cooled copper coils to a tailor‐made air‐cooled Litz‐wire coil (100 kHz, up to 70 mT) was found particularly beneficial to improve *E*_T_. In addition, the generation of eddy currents by the microparticulate heating agents was found to complement favorably the classical hysteresis heating mechanism. Overall, an *E*_T_ of ca. 5% was determined under optimized conditions (flow = 0.3 L h^−1^, *P*_coil_ = 70 W), with the potential to reach 18% at maximum theoretical efficiency of the set‐up used.

Amind and coworkers explored the impact of field frequency and coil geometry on the *E*_T_ of CoNi NPs‐catalyzed induction‐heated steam methane reforming.^[^ ^83^ ^]^ The authors compared the power consumption of the coil under operation with the power taken up by the reaction. They observed that increasing the ACMF frequency from 68 to 189 kHz allowed observing similar conversion levels (90% methane conversion) while consuming less power (500 W instead of 800 W), resulting in a higher *E*_T_ (18% vs. 10%). The geometry of the water‐cooled coil also had a noticeable influence, with long and narrow coils being more favorable than short and wide coils. As a result, the *E*_T_ could reach 23% in the optimized setup, as compared to 11% in the standard bench‐scale equipment.

Recently, Noble et al. reported on the theoretical modelling of a 20 cm diameter induction‐heated catalytic flow reactor for which the coil is placed inside the reactor wall.^[^ ^47^ ^]^ Taking the dehydration of ethanol to ethylene as a model reaction and a catalyst bed heated by a combination of hysteresis and eddy current heating, the authors calculated a maximum potential *E*_H_ of 65% (voltage of 11 kV, frequency of 10 kHz). Frequency of the ACMF and reactor scale (i.e., volume fraction of heating material) were found to affect *E*_H_ the most. For a reactor diameter above 1 m, the authors even predicted *E*_H_ up to 90%. This theoretical work suggests that magnetically induced catalysis is potentially highly energy efficient and suitable for practical application on industrially relevant scale, although experimental studies are required to investigate the validity of this model.

While scarcely and disparately determined, the energy efficiencies of experimental magnetically induced processes are promising and steadily improving over the years. In addition, the main parameters affecting *E*_H_ have been identified (e.g., nature of heating material and heating mechanism, coil geometry and cooling, ACMF amplitude and frequency, and reactor scale), paving the way toward the development of cheaper and more efficient future systems closing the gap with the potentially excellent energy efficiencies of model systems (65%–90%). For *E*_T_, the flow of reactants and products was shown to be dominating parameter at high GHSVs. Besides experimental efforts, additional modeling, life cycles, and systems implementation analyses will also be required for further evaluation of the potential of magnetically induced catalysis for practical application. To facilitate energy efficiency determination and facilitate comparisons between studies and technologies, we suggest systematically providing *E*_H_.

Besides *E*_H_ and *E*_T_, we introduce here the ***energy efficiency toward product formation E*_P_**, which refers to the amount of product that can be synthesized per unit of energy. It is particularly relevant when considering chemical products and chemical reactions in general and represents an easy‐to‐calculate parameter that can potentially facilitate comparison across catalytic technologies as output product formation, input power, and reaction time are universal and straightforward to monitor. The reaction pathway toward the target product must be the same across the catalytic technologies considered to ensure meaningful comparison of *E*_P_ values. It is defined as:

(3) $$E_{P}=\left(n_{\mathrm{Product}}\right)/\left(P_{\text{ACMF generator}}+P_{\text{Cooling system}}\right)\times t]\;\text{in mol}.J^{-1}$$

where *n*_Product_ is the quantity of target product generated in mol, *P*_ACMF generator_ is the power consumed by the ACMF generator (in W), *P*_Cooling system_ is the power consumed by the cooling system (in W), and *t* is the reaction time in seconds.

## 4 Part (III): Potential Opportunities with Magnetically Induced Catalysis

Besides the already “unlocked” benefits of localized, rapid, and energy‐efficient heating, other effects may arise from magnetically induced catalysis. The occurrence or relevance of these effects is, however, still to be fully evaluated and validated, hence requiring systematic investigations at the fundamental level.

### 4.1 Can Magnetically Induced Catalysis Enable Effective Energy Management?

While magnetically induced catalysis has been successfully used to enable endothermic reactions, its suitability in terms of energy efficiency still requires detailed evaluation. The localized constant heating of catalysts at high temperature in a colder environment is expected to be beneficial for both thermodynamics and energy efficiency, but experimental demonstrations are desired.

Magnetically induced catalysis also offers opportunities for very rapid “cold start” of exothermic reactions, as well as for pulse heating to maintain catalytic performance in reactions that are not fully thermally self‐sustained once started.

### 4.2 Can Magnetically Induced Catalysis Enable Novel Reaction Pathways and Processes?

**Figure 12:**
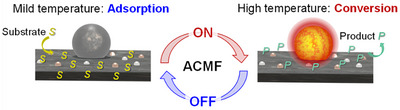
Potential of magnetically induced catalysis for rapid pulse substrate adsorption/conversion operation modes.

The rapid heating has so far been used to switch ON and OFF activity for adaptivity to intermittent power supply but may offer new opportunities to open new reaction pathways and rapidly tune the selectivity of transformations following consecutive and parallel pathways. For example, Lin et al. showed that Cu‐decorated ICNPs possess excellent hydrodeoxygenation activity under magnetically induced conditions, but remain only hydrogenation‐active under conventional heating irrespective of the applied temperature.^[^ ^75^ ^]^ Similarly, low pressure amide hydrogenation could be enabled by magnetically induced catalysis using ICNPs@Pt/Al_2_O_3_, while conventional heating failed to provide substantial conversion.^[^ ^65^ ^]^ Rapidly switching the temperature of catalytic systems may also allow for pulse operations modes (e.g., substrate adsorption facilitated at mild temperature and conversion at high temperature, as illustrated in Figure 12).

The generation of local hot spots in a cold environment may enable reactions or the connection of reaction steps in multistep approaches with transformations or processes involving temperature‐sensitive compounds (substrates, intermediates, products, catalytic species). The potential of this approach was recently highlighted by Varela‐Izquierdo et al. who reported the amination of alcohols with Co_4_Ni_6_@Cu NPs whereby the redistribution reactions typically occurring on amines at high temperatures were prevented under magnetically induced conditions.^[^ ^86^ ^]^

As another example, one could think of a heterogeneously catalyzed reaction enabled at the hot surface followed by an organometallic or enzymatic reaction step in the cold bulk solution (cf. concatenation^[^ ^94^ ^]^).

### 4.3 Can Magnetically Induced Catalysis Induce Magnetic and/or Electric Effects Relevant for Catalysis?

In electrocatalysis, the application of strong static magnetic fields was found to drastically promote the activity of magnetic catalysts toward various transformations (e.g., oxygen evolution reaction, CO_2_ reduction reaction) through mass transport effects and promotion of spin‐facilitated pathways.^[^ ^95^, ^96^, ^97^ ^]^ In magnetically induced catalysis, such effects are supposed to be negligible due to the oscillation of magnetic fields at high frequencies. However, besides the “simple” heating of magnetic catalysts, are there other potential magnetic effects occurring upon exposure to ACMFs that may influence the reactivity of catalysts?

Then, ACMFs generate eddy current in conductive materials, which may lead to significant heating depending on the geometry of the material. Besides this potential thermal energy generation, has the induced “electrification” of catalysts through eddy current any impact on observed catalytic performances? Of particular interest would be the investigation of the electric polarization of catalysts heated by ACMFs through the operando characterization of their electronic properties. Thorough investigation of these questions will require multidisciplinary experimental and theoretical efforts from physicists and chemists.

### 4.4 Can Magneto‐Catalysis Enable the Decoupling of Local and Bulk Equilibria?

Magnetic induction heating generates high temperatures on catalysts that are in colder environments, leading to a nonuniformity of temperatures in reactors that can result in different equilibrium boundaries at the surface relative to the bulk of the reaction medium. This requires nonadiabatic operation that can be achieved especially in continuous flow reactors, potentially providing opportunities for the decoupling of the kinetically relevant temperature from the bulk temperature in typically equilibrium‐limited exothermic reactions. For example, strong temperature gradients generated by active cooling were shown to be beneficial in conventional CO_2_ hydrogenation to methanol, pushing the reactions equilibrium and improving CO_2_ conversion and methanol selectivity.^[^ ^98^ ^]^ magnetically induced catalysis may provide comparable benefits, while obviating the need for active cooling. In this context, De Masi et al. showed that Fe_30_Ni_70_@Ni NPs immobilized on SiRAlOx could achieve a methane yield of 100% in the magnetically induced continuous flow hydrogenation of CO_2_ (19 mT, 300 kHz, *P*_atm_, H_2_:CO_2_ 4:1, 25 mL min^−1^) at a catalyst bed temperature of 350 °C as determined by a Pt thermocouple. Interestingly, this is substantially higher than the theoretical maximum methane yield at this temperature and conditions (ca. 85%).^[^ ^99^ ^]^

Nevertheless, it should be noted that the capability of magnetically induced catalysis to generate catalyst particles hotter than their surroundings may not be universal and can depend on reactor, catalyst bed, and flow conditions designs.

### 4.5 Can Magnetically Induced Catalysis Induce Localized High Pressure Around Hot Spots?

For magnetically induced catalysis in solution, the generation of high‐temperature hot spots in solvents of comparably low boiling points can build up a gaseous layer around magnetic catalysts (cf. Leidenfrost effect). This can potentially result in localized increase in the pressure around catalytically active sites, but also generate mass transport limitations. The increase of endogenous pressure in the cavities of Pt‐loaded iron oxide‐based hollow nanoreactors under an ACMF was recently proposed and investigated by Zhang and coworkers. The authors reported a pressure increase from 3 to ca. 10 bar in their tailor‐made nanoreactors during the hydrogenation of α,β‐unsaturatedaldehydes/ketones to unsaturated alcohols (60 mT, 100 kHz, in ethanol), and claimed it to be favorable for reaction kinetics as well as to prevent over‐hydrogenation.^[^ ^100^ ^]^ For the challenging hydrogenation of amides that requires higher catalyst surface temperatures, Lin et al. have shown the Leidenfrost effect to be severely limiting, with catalytic performance strongly influenced by the boiling point of the solvents used.^[^ ^65^ ^]^ The occurrence of such pressure build‐up effects and their impact on catalytic performance depend on the transformation and catalytic system used, and their identification and demonstration still require thorough investigation.

### 4.6 Can Magnetically Induced Catalysis be Scaled‐Up?

As described in the Introduction section, magnetic induction heating is a mature technology used for decades in metallurgy and biomedicine, and commercial industrial‐scale ACMF generators are thus readily available on the market, with a wide variety of possible coil sizes and shapes. The availability of ACMF generators thus does not seem to be a limitation toward industrial application of magnetically induced catalysis. One should keep in mind, however, that ACMF parameters and energy consumption strongly depend on the coil diameter, which is why typical coil diameters lie in the 1–50 cm range. Consequently, magnetically induced catalysis is believed to be best suited for continuous flow catalysis, where reactor sections remain moderate. Successful application of magnetically induced catalysis to gas phase and liquid phase continuous flow reactions has been demonstrated on lab‐scale equipment. While an industrial‐scale demonstration of a magnetically induced process has not been reported yet, several research teams and companies are currently working on the development of pilot plants. Operating under continuous flow also enables to utilize nonmagnetic reactor materials, obviously a prerequisite for magnetically induced catalysis. This is particularly relevant for reactions requiring elevated pressures that would be conventionally conducted in steel reactors. Different approaches are presently being studied including the use of polymeric, crystalline, or ceramic materials.

## 5 Conclusions and Outlook

The design and application of catalysts and materials that are susceptible to activation by magnetic fields and especially alternating current magnetic fields (ACMFs) is finding a rapidly growing interest. This perspective article provides a unifying definition for these approaches as ***magnetically induced catalysis*** and introduces a set of parameters to describe the techniques and document the data generated in corresponding research efforts in different laboratories. Thus, we hope to provide a coherent framework for the progress in this field to ensure validation of different approaches within the field, and in comparison to alternative techniques for electrified and/or adaptive catalytic systems.

The heating agent can be the catalyst itself as a nanoparticle or the thermal activation is transferred from deposited heating agents to the active site or supported metal NPs. Alternatively, catalysts can be heated by NPs or a material (e.g., iron wool) applied externally, e.g., in physical mixtures. This shows the diversity of the approaches leading to magnetically induced catalysis and suggests that many more ideas will be exploited in the future. The selected examples covering gas phase as well as liquid phase reactions demonstrate the generation of strong temperature gradients by magnetic catalysts heated by ACMFs, and illustrate the main associated benefits. In particular, selective and localized catalyst heating has the potential to save energy, to enable high temperature transformation in overall colder environments, and to allow unusual tandem reactions by taking advantage of temperature gradients. This is profitable for working with thermally sensitive molecules or with different catalysts working at different temperatures. Of particular interest is the capacity of magnetic induction activation to enable challenging transformations under very mild observable conditions of pressure and temperature, which may provide new opportunities for the practical implementation of these reactions in research and industry. The induced temperature gradients may also open the possibility to work in out‐of‐equilibrium conditions or more precisely in the presence of different equilibria depending on the temperature. The fast heating upon application of the ACMF and the rapid cooling immediately when it is switched off enable reversible switching activity ON and OFF. While not yet demonstrated up to now, it is certainly conceivable to use this effect also to switch between two stages of different selectivities for a given catalyst. Adapting catalysts to intermittency in energy supply is just one of the various possibilities associated with such a scenario.

While these effects are equally fascinating and practically useful, they represent only a small fraction of the landscape of magnetically induced catalysis yet to be explored. We hope that the conceptual overview in this article provides the basis for rapid progress in this exciting field and stimulates further research activities to evaluate and validate the associated opportunities.

## Conflict of Interests

The authors declare no conflict of interest.

## Data Availability

Data sharing is not applicable to this article as no new data were created or analyzed in this study.
